# Evidence for objects of implementation in healthcare: considerations for *Implementation Science* and *Implementation Science Communications*

**DOI:** 10.1186/s13012-022-01249-w

**Published:** 2022-12-16

**Authors:** Michel Wensing, Anne Sales, Gregory A. Aarons, Dong (Roman) Xu, Paul Wilson

**Affiliations:** 1 Heidelberg University Hospital, Heidelberg, Germany; 2 Sinclair School of Nursing and Department of Family and Community Medicine, University of Missouri, Columbia, MO USA; 3 Center for Clinical Management Research, VA Ann Arbor Healthcare System, Ann Arbor, MI USA; 4 Department of Psychiatry and ACTRI Dissemination and Implementation Science Center, University of California San Diego, La Jolla, San Diego, CA USA; 5 SMU Institute for Global Health (SIGHT), School of Health Management and Dermatology Hospital , Southern Medical University (SMU), Guangzhou, China; 6 Centre for Primary Care and Health Services Research, University of Manchester, Manchester, UK; 7 NIHR Applied Research Collaboration Greater Manchester, Manchester, UK

## Abstract

The journals *Implementation Science* and *Implementation Science Communications* are focused on the implementation of evidence into healthcare practice and policy. This editorial offers reflections on how we handle this as editors. Studies that focus on the simultaneous implementation of implementation objects and (technological or other) structures to enable their implementation are considered on a case-by-case basis regarding their contribution to implementation science. Studies on implementation objects with limited, mixed, or of out-of-context evidence are considered if the evidence for key components of the object of interest is sufficiently robust. We follow GRADE principles in our assessment of the certainty of research findings for health-related interventions in individuals. Adapted thresholds apply to evidence for population health interventions, organizational changes, health reforms, health policy innovations, and medical devices. The added value of a study to the field of implementation science remains of central interest for our journals.

## Introduction

The journals *Implementation Science* and *Implementation Science Communications* focus on the implementation of evidence into practice and policy. The evidence concerns objects of implementation, such as clinical or public health interventions, guidelines, medical technologies (medicines, devices), and healthcare delivery models (e.g. structured diabetes care). We have never operationalized the concept of evidence in healthcare in detail, but in the journal editorial that launched *Implementation Science*, Eccles and Mittman made it clear that it relates to research evidence rather than evidence from practical experience or other sources [1]. There are multiple related terms commonly used in healthcare, including evidence-based practice, evidence-based intervention, and evidence-based medicine. Even the concept “practice-based evidence” implies the need for garnering rigorous evidence of the value of practice-based experience. The assumption underlying these terms is that practices, programmes, devices, or innovations more generally need to be shown to have benefits, and no unacceptable harms, before these are widely implemented in practice or policy. Our working operationalization of evidence in healthcare has included well-developed, trustworthy clinical practice guidelines [2], systematic reviews and meta-analyses of primary studies [3], and, in rare instances, single rigorous studies (typically randomized trials) of the practice of interest. It is incumbent on study authors to be clear about the research evidence supporting the “thing” [4] or “implementation object” that is being implemented and how that was assessed. Some submissions are rejected, because the thing being implemented lacked a sufficient evidence base or was poorly operationalized and assessed.

However, some researchers perceive that this threshold is not entirely explicit, difficult to reach, or inappropriate in certain contexts. Our journals will remain focused on the implementation of evidence-based practices, programmes, or policies in healthcare, but we believe that there are several reasons to reflect on what is meant by this. In many cases, a set of practices is implemented, some of which are clearly evidence-based while others are not, or have mixed evidence. For instance, many clinical practice guidelines contain recommendations with a strong evidence base and recommendations with weak or conflicting evidence. Furthermore, using guidelines requires clinical judgement, ideally in partnership with patients [5]. Second, research evidence needs to be understood in context, so the transportability of research findings from one setting to another can be an issue [6]. For instance, is research in the USA relevant for implementation in Africa or Asia? Or is evidence based on clinical trials with predominantly White populations also evidence based for Hispanic or Indigenous populations? Third, some practices depend on the presence of technical, legal, and other arrangements, which are interventions in their own right that need to be created before the practices can be evaluated for benefits and harms. For instance, health-related digital devices depend on information technology infrastructures, and advanced nursing roles require educational programmes and regulatory arrangements. In these situations, developing the necessary structures precedes the generation of evidence on intervention outcomes. Fourth, models of “precision medicine” or “precision healthcare” may imply the simultaneous conduct of patient care, research, and decision support. For instance, genetic profiling of individual patients can guide their medical treatment in oncology while simultaneously generating data for research and decision support. This challenges the assumption that evidence generation precedes its implementation. Fifth and last, we observe that strong research evidence (i.e. from randomized trials) is not always perceived to be required or appropriate. For instance, several health authorities require professional assessment, but not necessarily clinical trials, for approval of some interventions (e.g. medical devices and healthcare delivery models). This implies that such interventions, particularly those with low risk, are approved for use, although they are not evidence based through randomized trials.

In the remainder of this commentary, we will discuss three specific cases and how we deal with these in our journals. Before we turn to this, we summarize the arguments for a focus on evidence-based practices for implementation.

### Why is evidence important?

Our rationale for a focus on the implementation of evidence-based practices, programmes, devices, and policies (rather than those of unproven value) is linked to the argument for the use of evidence in healthcare generally. Historically, it implies a departure from authority-based practice, which was motivated by examples of outdated practices that continued to be used and new practices of proven value that were only implemented after many years, or not at all [7]. The use of evidence to guide healthcare practice has become a broadly accepted ideal. Several decades after the introduction of evidence-based healthcare, we perceive that many innovations are implemented in healthcare practice or policy that have unproven value or proven lack of value, require resources, and may cause harm. For example, there is debate regarding benefits and harms of healthcare practices such as breast cancer screening [8] and HIV self-testing [9]. Also, the majority of new medical technologies used in German hospitals were not supported by convincing evidence for their added value [10], which may not be the best use of resources to optimize health and well-being of individuals and populations.

Implementation heavily focuses on implementation strategies, whose choice, optimization, and effectiveness require dedicated research. Furthermore, the field examines the context(s) in which an object is implemented, as the application of objects does not happen in a vacuum but involves complex interactions with many contextual factors (such as service systems, organizations, clinical expertise, patient preferences, and available resources). Nevertheless, here we focus on the evidence for the objects of implementation, which also influence the uptake of objects into practice. Beginning with the ground-breaking work of Everett Rogers [11], factors related to the object being implemented—the innovation, intervention, evidence-based practice, and so on—have been theorized to be critical in influencing the success of implementation efforts. Since Rogers’ work, most consolidated determinant frameworks (e.g. [12–16]), as well as many others derived from empirical and theoretical approaches, have included domains related to factors related to the thing being implemented, including the perception of the strength of evidence. In Rogers’ model, effectiveness of the innovation is seen to be influential in users’ decision to adopt the innovation, along with several other factors related both to the innovation and to the adopter of the innovation.

## Case 1: Implementation before or simultaneously with evaluation of effectiveness

Some interventions can only be applied and evaluated after specific technological, organizational, legal, or financial structures have been put in place. For instance, health-related software applications depend on technological infrastructure. The establishment of such new structures can be an intervention on its own, which may be evaluated in terms of benefits and harms. These typically facilitate various objects or practices being implemented, not just a particular one. Studies that entirely focus on implementing a technological or other infrastructure remain out of scope for our journals. In the context of *implementation science*, changes in structures (ERIC: change physical structure and equipment or change record systems; TDF/BCTT: restructuring the physical environment or restructuring the social environment; EPOC: changes to the physical or sensory healthcare environment) may be conceptualized as implementation strategies [17, 18]. Some studies examine the effects of an intervention, for which changes in technological or other structures were made. If the object of implementation is not evidence based, an evaluation study will be a hybrid effectiveness-implementation study type 1 (i.e. emphasis is on clinical effectiveness) [19]. So far, we have excluded such designs from the scope of our journals, because they are not primarily designed to examine aspects of implementation and tend to be descriptive regarding implementation aspects. However, we have decided to consider such studies on a case-by-case basis, considering the substance of their contribution to the field of implementation science. Purposive analysis, rather than just application and description of tests of implementation strategies in context, implementation concepts, or implementation frameworks, is required to have value for the field of implementation science. Factors that further increase the relevance of studies for implementation science include proximity to real-world practice, generalizability regarding healthcare or other settings (i.e. multisite studies), and rigorous study design, such as those involving randomization (or allocation) of clusters of participants to study arms (rather than individually randomized trials) to reduce contamination of implementation strategies [20].

## Case 2: Limited, mixed, or out-of-context evidence

In many situations, the research evidence related to the implementation object is limited, mixed, or out of context. Examples are the treatment of post-Covid syndrome (limited evidence), case management of chronic disease (mixed evidence), and decision aids for patients (potentially out of context for low-income countries). Other examples are diagnostic tests that have been examined for predictive value, but not regarding clinical effectiveness in a targeted population. While we cannot provide pertinent requirements, the following provides some guidance.

In all cases, we prefer a consolidated synthesis of studies on interventions (i.e. systematic review) over evidence from single studies. If interventions imply substantial risks, costs, or consequences for health equity, the synthesized research evidence is required to be strong, coherent, and relevant to the context of the application. Ideally, the evidence relates to the primary active ingredients of the package that are implemented. Complex interventions or other multiple component interventions may need further justification or optimization for use in a given context if their effectiveness varies substantially across trials despite the pooled overall evidence. Complex interventions may need further testing if they are adapted after the effectiveness research. Recommendations for practice or policy should be accompanied by reflections on research limitations, heterogeneity, and context. Our expectations regarding the required strength of evidence are discussed in the subsequent section.

## Case 3: New perspectives on strength of evidence

Thresholds for quality, strength, or certainty of research evidence are a topic of debate and development. In the context of systematic reviews and clinical practice guidelines, the GRADE principles [21] have been widely adopted and extensively documented. An extension to qualitative research is available [22]. The GRADE approach provides a middle ground between the belief that certainty of evidence is primarily related to study designs (randomized trials versus observational designs) and the belief that it requires a detailed assessment of many specific methodological features. More specifically, GRADE proposes that a well-conducted randomized trial provides high certainty, which is downgraded if its execution causes risk of bias. On the other hand, an observational evaluation design provides low certainty, which is, however, upgraded if it is well executed and finds large intervention effects. GRADE recognizes, particularly in relation to public health interventions, that there are differences in perspectives and in cultures of evidence that can impact evidence thresholds necessary to support decision-making [23]. We recognize this too, and the GRADE approach reflects our journals’ expectations regarding clinical and other health-related interventions that target individuals or small groups.

### Population health interventions

We remain interested in the implementation of population health interventions mediated through agencies, technologies, or networks, typically involving healthcare providers but potentially others—such as interventions like community health workers who connect people to health and care resources in the community or child welfare workers who engage families in behavioural health services. We welcome evaluation via randomized designs, natural experiments, rigorous quasi-experimental designs, and other designs. However, we realize that interventions that target populations may not be examined in randomized trials or related designs. We are willing to accept population health interventions as implementation objects, if these were evaluated in studies that provided the highest level of certainty under the given circumstances. We recognize that non-randomized and or “natural” experiments and other designs can provide adequate evidence when randomized designs are not possible, where interventions are demonstrably feasible and acceptable, and where there is little potential for harm [24]. Features such as comparison arms, repeated measurements, adequate analysis to adjust for potential confounders, and sensitivity analyses contribute to the trustworthiness of outcome evaluations. We note that we expect a clear justification and thoughtful discussion on these points when this is the basis of the existing evidence for an implementation object.

### Organizational changes, health reforms, and health policy innovations

Large-scale service delivery reconfiguration to improve healthcare and health outcomes is an area that generally does not lend itself to randomized designs. Nevertheless, we have published on the implementation of such changes or policies in the past and will continue to do so. Similar to population health interventions, we would accept organizational changes and health policies that are examined using evaluation designs that optimize the certainty of findings. For example, a study that seeks to improve the reach of an evidence-based clinical intervention through targeting organizational culture or climate must address the degree to which the change affected the reach and/or outcomes of the clinical intervention. It is also critical that the theory of change be tested and its relation to the appropriate theory, model, or framework also be clearly articulated. That is, the causal pathways between determinants, mechanisms, and outcomes should be considered and tested to the extent possible.

### Medical devices

Medical devices comprise an emerging field of research and development, covering a broad range of tools varying from wheelchairs and surgical materials to sensors for home care and health apps for patients. If such devices imply substantial risk of harm, or high cost, they should be examined in clinical trials in order to be considered evidence based [25]. We do not publish on the implementation of non-approved medical devices in the context of clinical or public health trials. Some of these devices (e.g. blood pressure or heart rate monitors) are supposed to have little risk of harm, but health authorities have heterogeneous arrangements for approval. In the USA, the Food and Drug Administration maintains a rigid mandate for randomized clinical trials to demonstrate both efficacy and some threshold of safety prior to approving therapeutic agents or medical devices. On the other hand, in the European Union, medical devices of lower risk are required to prove safety but are exempted from the need to provide evidence from clinical trials. Approval of a medical device does not automatically imply reimbursement in a health insurance system. For medical devices, we will make a case-by-case assessment of devices where there is evidence that they have been approved by a relevant jurisdictional authority.

## Conclusions

We fully appreciate that the discussion in this editorial only covers issues pertaining to submissions to the journals *Implementation Science* and *Implementation Science Communications*. There is an entire field of study in philosophy and related areas devoted to the nature of evidence, and a similar discussion in areas outside healthcare can certainly be engaged. We have attempted to clarify a complex topic that arises frequently as a problem in reviewing manuscripts submitted to our journals.

## Data Availability

Not applicable
